# Synergies between Heat Disturbance and Inoculum Size Promote the Invasion Potential of a Bacterial Pathogen in Soil

**DOI:** 10.3390/microorganisms10030630

**Published:** 2022-03-16

**Authors:** Xin Gong, Ziyun Zhang, Hui Wang, Huixin Li, Feng Hu, Manqiang Liu, Lin Jiang, Xiaoyun Chen, Chao Ma

**Affiliations:** 1 Soil Ecology Lab, College of Resources and Environmental Sciences, Nanjing Agricultural University, Nanjing 210095, China; xgong@njau.edu.cn (X.G.); seven132@163.com (H.W.); huixinli@njau.edu.cn (H.L.); fenghu@njau.edu.cn (F.H.); liumq@njau.edu.cn (M.L.); 2 Anhui Province Key Laboratory of Farmland Ecological Conservation and Pollution Prevention, Key Laboratory of JiangHuai Arable Land Resources Protection and Eco-Restoration, College of Resources and Environment, Anhui Agricultural University, Hefei 230036, China; ziyunjiayou@163.com; 3 School of Biological Sciences, Georgia Institute of Technology, Atlanta, GA 30332, USA; lin.jiang@biology.gatech.edu

**Keywords:** disturbance, global change, microbial invasion, propagule pressure, resource availability

## Abstract

Inoculum size contributes to the invasion potential of pathogens in the soil. However, the role of inoculum size in determining the fate of pathogens in disturbed soils remains unclear. Herein, we investigated the survival rates of a bacterial pathogen, *Ralstonia solanacearum*, in soils subjected to heat as a simulated disturbance. Our results revealed that heating increased soil resource availability but reduced resource differentiation between *R. solanacearum* and indigenous bacterial communities. In both non-heated and heated soils, invader abundances increased with inoculum size, with a greater magnitude in heated soils. Inoculum size and heat-induced increases in soil-available carbon and nitrogen best predicted invasion success. Altogether, our findings suggested that the invasion by soil pathogens could be predicted by synergies between heat perturbation and inoculum size.

## 1. Introduction

A well-accepted aspect of global climate change is the increase in both the frequency and magnitude of extreme climate events, such as heat waves [1]. Climate extremes can promote pathogen invasion by facilitating the transport of propagules into new regions and decreasing the resistance of indigenous microbial communities to exotic invaders [2]. However, the role of heat wave-induced disturbance in the invasiveness of soil-borne pathogens to healthy soils remains poorly understood. In addition, increasing empirical evidence has suggested that the size of the introduced inocula is vital in determining the invasion success of alien species [3,4]. Alien pathogens with larger inoculum sizes are more likely to invade successfully than species with smaller inoculum sizes, as the population growth of many introduced species with small inoculum sizes tends to be negative, making these species vulnerable to extirpation; this is summarized by the Allee effect [5].

*Ralstonia solanacearum* belongs to the bacterial phylum *Proteobacteria* [6]. It is a soil-borne pathogen that infects plant roots and specifically invades the xylem vessels. It is the causal agent of global bacterial wilt disease, causing 50 to 100% yield reductions in more than 100 economically important crops [7]. Global climate change might elevate the risk of R. *solanacearum* invasion into healthy soils [8]. A vegetable–rice rotation is often used to prevent the wilt disease caused by *R. solanacearum* [9]. Occasionally, *R. solanacearum* has been reported to survive in rice paddy soils with rotation systems [10,11]. The potential invasion risk of *R. solanacearum* to paddy soils is likely to be elevated under future scenarios of climate change [12], threatening the health of these soils. For instance, heat waves might modify the availability of nutrients [13], which determines the invasiveness of *R. solanacearum*.

**Figure 1:**
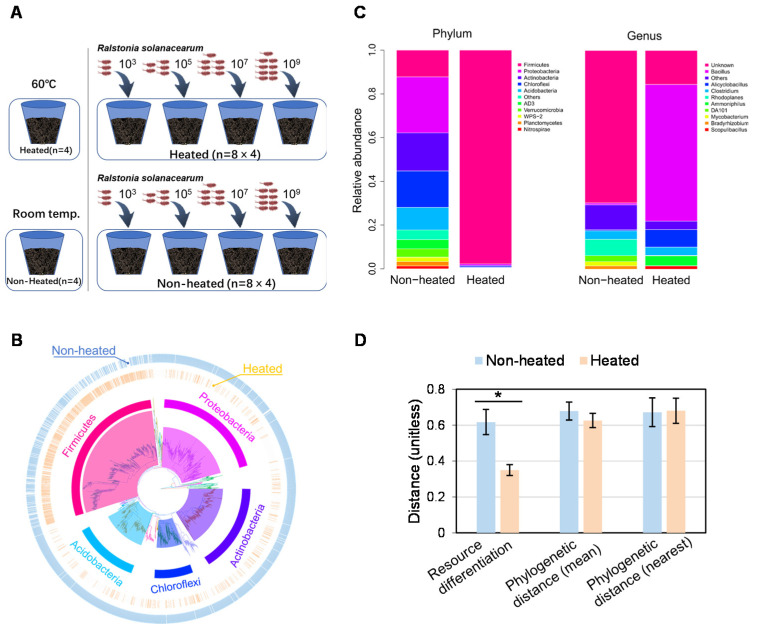
Schematic of the experimental design and the effects of heating on the characteristics of the pathogen and the indigenous soil bacterial community. The pathogen *R. solanacearum* was added to the soil, which was heated or not, at the indicated inoculum sizes, i.e., forming final concentrations of 10^3^, 10^5^, 10^7^, and 10^9^ CFU·g^−1^ dry soil (**A**). The phylogenies (**B**) and community composition (**C**) of bacterial communities in non-heated and heated soils. A heatmap showing the presence/absence of the tip in non-heated and heated soils has been appended at the outer and inner ring, respectively, in Panel C. The resource differentiation was calculated as the difference in carbon utilization substrates in Biolog, and the phylogenetic relatedness (the mean and the nearest phylogenetic distance calculated using the phylogenetic tree) between *R. solanacearum* and indigenous bacteria (**D**). Values are presented as means ± sd, * representing significant differences between non-heated and heated soils (*p* < 0.05, *t-*test).

Here, we conducted a microcosm experiment to explore the response of *R. solanacearum* after being inoculated into paddy soils with and without heating. Briefly, 72 microcosms were established by weighing 80.0 g soil into 250 ml serum bottles. Half of the microcosms were subjected to 60 ℃ for 24 hours as a heat treatment, and the other half were left at room temperature as controls. Next, the soil’s biological and physicochemical properties were analyzed after 3 and 42 days of incubation (Figure 1A). We hypothesized that heating would reduce the abundance of bacteria and bacterivores in the indigenous communities while simultaneously increasing resource availability, thereby promoting the positive effect of inoculum size on the invasion success of *R. solanacearum.*

## 2. Materials and Methods

### 2.1. Soil

The soil, classified as Acrisols and Ferralsols in the FAO classification system, was collected from a rice paddy field at the Red Soil Institute of Jiangxi Province, China (116°20′24′′ N, 28°15′30′′ E). The soil contained 26.6 g kg^−1^ total organic C, 2.6 g kg^−1^ total N, 24.8 mg kg^−1^ available N, pH 5.3, 84% sand, 12% silt, and 4% clay. Before the onset of the microcosm experiment, the cultivated layer (0–15 cm) was collected from the field and passed through a 5 mm sieve to remove the stones, large root fragments, and soil fauna. The soil was then adjusted to 70% water holding capacity, followed by incubation in darkness for 2 weeks at 22 °C, with frequent monitoring of the emission rates of CO_2_. 

### 2.2. The Bacterial Strain Used as an Invader

The strain was an RFP-tagged *Ralstonia solanacearum* (*Ralstonia solanacearum* QLRS1115-rfp), supplied by the National Engineering Research Center for Organic-based Fertilizers, Nanjing, China [14]. *R. solanacearum* is a soil-borne Gram-negative bacterium that infects plant roots and multiplies in the cortical tissue before entering the xylem [15]. In a matter of hours after infection, the bacteria spread into the crown and stem, causing wilt, generalized necrosis, and plant death. After RFP tagging, the phenotypic, physiological, and biochemical traits of the experimental bacteria did not show significant differences from its parent strains [16].

### 2.3. Experimental Design and Setup

Heat waves frequently occur in the study area, with the land surface temperatures increasing from year to year. The maximum temperature has exceeded 60 °C (Figure S1), we thus chose 60 °C for the heating disturbance. Seventy-two microcosms were established by weighing 80.0 g soil into 250 ml serum bottles. All the bottles were sealed with a rubber plug, and then half of the microcosms (36 bottles) were subjected to heating at 60 °C for 24 h (i.e., the disturbed treatment) and the other half (36 bottles) were left at room temperature (i.e., the non-disturbed treatment). All microcosms were then unsealed and vented in a sterile fume hood for 2 h at room temperature to return the microcosms to ambient conditions (e.g., temperature and CO_2_ production). Four replicates of the disturbed and non-disturbed microcosms (i.e., 8 bottles) were destructively sampled to measure the soil resource and community properties. 

Before inoculation, *R. solanacearum* was cultured by shaking in 50 ml LB liquid medium (220 rpm, 30 °C) supplemented with gentamicin (30 µg ml^−1^), which was used to promote RFP protein expression and suppress non-target bacteria. At the mid-exponential growth stage, cells were washed 3 times by centrifugation (8000× *g* for 5 min) and re-suspended in sterile ultra-pure water to a serial gradient of 10^4^, 10^6^, 10^8^, and 10^10^ CFU·g^−1^ dry soil. Next, 3 ml aliquots of the bacterial suspension were inoculated to the remainder of the microcosms (64) by gentle pipetting and mixing to establish a final cell density of 10^3,^ 10^5^, 10^7^, and 10^9^ CFU·g^−1^ dry soil (Figure 1A).

### 2.4. Measurements

The population size of *R. solanacearum* in soil was determined by plating serially diluted soil samples onto LB agar plates with gentamicin and counting the number of fluorescent colonies at appropriate dilution levels on a UV transilluminator (LB-16, Maestrogen, Las Vegas, NV, USA). Soil pH was measured using a pH meter (FP20, Mettler Toledo, Greifensee, Switzerland), and EC (electrical conductivity) was measured using an EC meter (C3010, Consort, Turnhout, Belgium) in a 1:5 soil-to-water ratio [17]. Dissolved organic C and N were extracted by sterile ultra-pure water and determined with a total organic carbon analyzer (Vario TOC, Elementar, Langenselbold, Germany) and the semi-micro Kjeldahl method, respectively [18]. Inorganic nitrogen was extracted with 2 M KCl and measured by continuous flow auto-analysis (AA3, Seal, Norderstedt, Germany). Protozoa were enumerated with a modified version of the most probable number method [19]. Bacterial biomass was assessed by colorimetrical quantification of phospholipid-phosphate after extraction by a chloroform–methanol–phosphate buffer mixture (1:2:0.8, *v*/*v*/*v*) and isolation from other lipids on silicic acid. The metabolic fingerprint of *R. solanacearum* and the soil microbiota was determined by the Biolog ECO plate (Biolog Inc., Hayward, CA, USA). To detect the phylogenetic relationship between the bacterial pathogen and the local bacterial community, we applied high-throughput sequencing analysis of the 16S rRNA gene within the V4 region, as recommended by EMP (https://earthmicrobiome.org/protocols-and-standards/16s/; accessed on 29 January 2022), after DNA extraction with the FastDNA SPIN Kit for Soil (MP Biomedicals, Solon, OH, USA). Universal primers 515F (50-AYTGGGYDTAAAGNG-30) and 802R (50-TACNVGGGTATCTAATCC-3) were selected for PCR amplification of the target region. Sequencing was conducted by Personal Biotechnology Co., Ltd. (Shanghai, China) using an Illumina MiSeq platform. For quality filtering and pair-end read assembly, pyrosequencing data were processed using the software Mothur version 1.27.1 [20]. Quality control steps were as follows: first, assembled contigs without an exact match to one of the sample barcode sets or primers were discarded; second, the remaining sequences were clustered into operational taxonomic units (OTUs) with the UPARSE algorithm, using the “-cluster_otus” command in USEARCH, based on a 97% similarity threshold, with chimera sequences identified and eliminated during the procedure. Sequences found only once across all samples were treated as singletons and removed in subsequent analyses. Taxonomic classification of each OTU was determined using the Ribosomal Database Project (RDP) classifier with a confidence threshold of 0.8 against the Greengene version 13_8 database. OTUs not classified into bacteria were removed. The samples were rarefied to 15,000 sequences per sample in the following analysis.

### 2.5. Statistical Analyses

The data were tested for normal distribution and homogeneity of variance by the Kolmogorov–Smirnov test and Levene’s test, respectively. The pairwise two-sample t-test between the non-disturbed and disturbed treatments was used for each of the soil properties. For the pyrosequencing data, the percentage of each taxonomy was designated as the relative abundance. Dose–response curves between propagule pressure and the invasion potential of non-indigenous bacteria were fitted using polynomial models via Origin 8.5 software (Microcal Software, Northampton, MA, USA). Two-way ANOVA was carried out to examine the main and interactive effects of propagule pressure and disturbance on the survival of non-indigenous bacteria in the soil. Strain survival on each treatment of two sampling dates was tested by one-way ANOVA followed by Duncan’s test. Pearson correlation analysis was performed to reveal the relationship between the survival of non-indigenous bacteria and the soil environment, resources, and community properties. Classification Random Forest (RF) analysis with 5000 permutations was performed to evaluate the most important predictors for the survival rates of *R. solanacearum* using the R packages randomForest, A3, and rfPermute version 2.1.81 [21,22,23]. All data are presented as non-transformed means (*n* = 4) and their standard deviation. For the construction of a phylogenetic tree, the QIIME script “make_phylogeny.py” with the default setting “FastTree” was used. Visualization of the phylogenetic tree was then implemented using the function ggtree in R [24,25]. Resource differentiation between *R. solanacearum* and the indigenous bacterial community was calculated as the distance of resource utilization between *R. solanacearum* and the indigenous bacterial community using the Biolog data. The mean and nearest phylogenetic distance was calculated as the phylogenetic distance between *R. solanacearum* and the indigenous bacterial community using the functions comdist and comdistnt, respectively, in R [26].

## 3. Results and Discussion

The variation of the bacterial community in the heated soil was accompanied by changes in the physicochemical properties. The concentrations of dissolved organic carbon (DOC), dissolved organic nitrogen (DON) and inorganic nitrogen in the soil were significantly increased in the heated samples (Supplementary Figure S2c–e). Furthermore, the decreased abundance of protists in the indigenous communities suggested that predation by microbivores was reduced in the heated soils (Supplementary Figure S2f). While bacterial diversity had decreased, the biomass of indigenous bacterial communities was not changed by heating (Supplementary Figure S2g). This result indicates that certain heat-resistant groups, mostly originating from the genus *Bacillus* in the phylum Firmicutes, proliferated in heated soils (Figure 1B,C). These fast-growing groups in indigenous communities might compete for the realized niches in heated soils against *R. solanacearum* [27]. Moreover, resource differentiation, estimated as the difference between the carbon utilization of *R. solanacearum* and that of the indigenous communities, was lower in the heated soils than in the non-heated soils (Figure 1D). Therefore, *R. solanacearum* faced decreased predation but increased competition for available resources with indigenous communities in heated soils [28].

**Figure 2:**
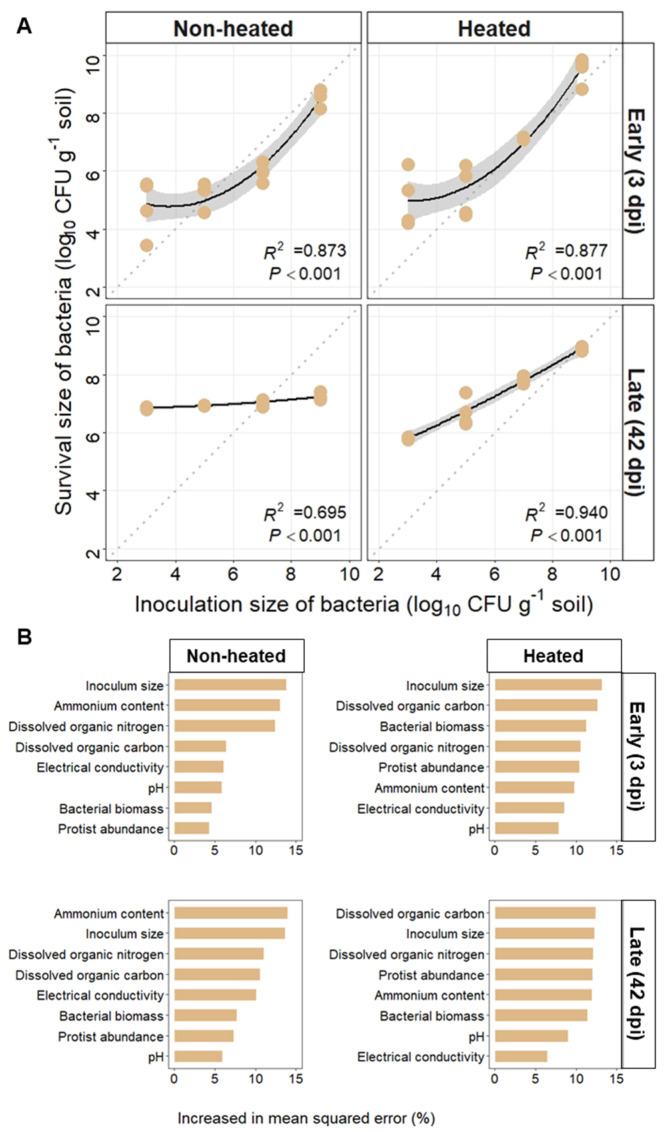
Dose–response curves for the survival of *Ralstonia solanacearum* in non-heated and heated soils at early (3 days post-inoculation, 3 dpi) and late (42 days post-inoculation, 42 dpi) stages; the values are log-transformed in the main figure (**A**). Shaded areas represent the 95% confidence intervals. The predictors for the survival of *R. solanacearum* in non-heated and heated soils at early (3 days post-inoculation, 3 dpi) and late stages (42 days post-inoculation, 42 dpi) were estimated by Random Forest, with the bars representing the increased percentage in the mean squared errors (**B**).

The larger inoculum size of *R. solanacearum* survived better in heated than in non-heated soils on both Days 3 and 42 after being introduced into the paddy soils (Figure 2A). Heating increased the slope of the dose–response curves of *R. solanacearum,* and the equilibrium dose increased from 10^7^ to 10^9^ CFU g^−^^1^ dry soil during the later stages. In non-heated soils, *R. solanacearum* with inoculum sizes lower than 10^7^ CFU g^−^^1^ dry soil, tended to increase to the equilibrium dose, i.e., 10^7^ CFU g^−^^1^ dry soil. In heated soils, the equilibrium dose was 10^9^ CFU g^−^^1^ dry soil, suggesting that more niches were available to *R. solanacearum* in the heated soils [29]. Furthermore, *R. solanacearum* is a pathogen capable of infecting a variety of plant species, and its abundance can predict the severity of bacterial wilt disease outbreaks [30]. The greater survival rates of *R. solanacearum* in heated than in non-heated soils suggest that the risk of disease incidence is elevated in heated soils such as those found under extreme hot weather conditions. Heating would increase the supply of available resources for *R. solanacearum*, which might support the multiplication of *R. solanacearum* at low inoculum sizes. However, survival rates at an inoculum size of 10^3^ CFU g^−^^1^ dry soil were not higher in heated than in non-heated soils, when interactions among the indigenous community are likely to be a factor influencing the survival of the alien *R. solanacearum*. Although heating-induced habitat changes can increase the risks of *R. solanacearum* invasion, invasions can be controlled if the inoculum size is less than 10^3^ CFU g^−^^1^ dry soil.

Inoculum size and heat disturbance influenced the available resources (DOC and ammonium) and were the dominant drivers of *R. solanacearum* survival in the early and late stages, respectively (Figure 2B). In the early incubation stages, inoculum size contributed to the survival rates of *R. solanacearum*. Allee effects, which indicate positive density dependence, suggest that fitness increases with increasing population density due to the benefits associated with the presence of conspecifics [31]. The benefits for invaders from the addition of successive inoculum sizes outweigh the costs, such as competition among conspecifics for resources, which are abundant in the early incubation stages. Thus, a net gain in fitness for successful invasion of paddy soil contributed to the survival of *R. solanacearum* [32]. In the late stages, the amount of *R. solanacearum* reached or nearly reached the carrying capacity of the habitats in both the non-heated and heated soils, making available resources more important than inoculum size in determining the survival of *R. solanacearum* [33]. Inoculum size thus interacts with other factors, such as the amount of available resources and the community composition of the invaded habitat [34]. Reducing the inoculum size is thus an effective approach to lowering the invasiveness of alien species [4], yet the development of effective management to stop new invasions by *R. solanacearum* seldom considers inoculum size [9]. Hence, our study provides a unique dataset that is both essential and practical. It can be used to monitor the relationship between inoculum size and indigenous habitats, and to manage soil-borne pathogen invasions in the soil. Future studies should pay more attention to detecting the interactions between *R*. *solanacearum* and indigenous microbes (e.g., *Bacillus*) as an example of controlling pathogen invasions and the biocontrol of wilt disease.

## 4. Conclusions

Heat disturbances enhanced the effects of inoculum size in predicting *R. solanacearum* invasions in soil, and this was attributed to the increasing available niches of resource dimensions in disturbed habitats. Furthermore, the equilibrium dose of *R. solanacearum* was determined to be 10^7^ CFU g^−^^1^ dry soil in paddy soils, but the value increased due to heating disturbance. Moreover, the survival of alien species was characterized by Allee effects; therefore, the invasion risk of *R. solanacearum* can be controlled if the inoculum size is under 10^3^ CFU g^−^^1^ dry soil. Heating increased the competition for resources instead of niches between *R. solanacearum* and indigenous bacteria. This result can be used to formulate control strategies under global climate change, where heat waves may increase the release of available resources in the soil and simultaneously increase the competition for resources between *R. solanacearum* and the indigenous bacterial community. 

## Data Availability

Amplicon sequences were deposited in NCBI with the accession number SRP111087.

## Supplementary Materials

The following supporting information can be downloaded at: https://www.mdpi.com/article/10.3390/microorganisms10030630/s1. Figure S1: Average and maximum land surface temperature of the sampling site spanning the period from 2002 to 2006. The data were provided by the National Ecosystem Science Data Center, National Science and Technology Infrastructure of China (http://www.nesdc.org.cn; accessed on 29 January 2022). Figure S2: Soil physicochemical and bacterial properties, including pH, electrical conductivity (us/cm), dissolved organic carbon (mg kg^−1^), dissolved organic nitrogen (mg kg^−1^), inorganic nitrogen (mg kg^−1^), protozoa (log cells g^−1^), and bacterial biomass (nmol g^−1^), in non-heated and heated soils before inoculating the non-indigenous bacteria (mean ± SD). The pairwise two-sample t-test between disturbed and non-disturbed soils was used. * *p* < 0.05 and ** *p* < 0.01.
