# Supplementary material for: Synergies between Heat Disturbance and Inoculum Size Promote the Invasion Potential of a Bacterial Pathogen in Soil
Source: Microorganisms. 2022 Mar 16;10(3):630. doi: 10.3390/microorganisms10030630 (PMC8950789; doi:10.3390/microorganisms10030630)
Supplement: Supplementary file 1 [file microorganisms-10-00630-s001.zip › microorganisms-1598419-supplementary.pdf]

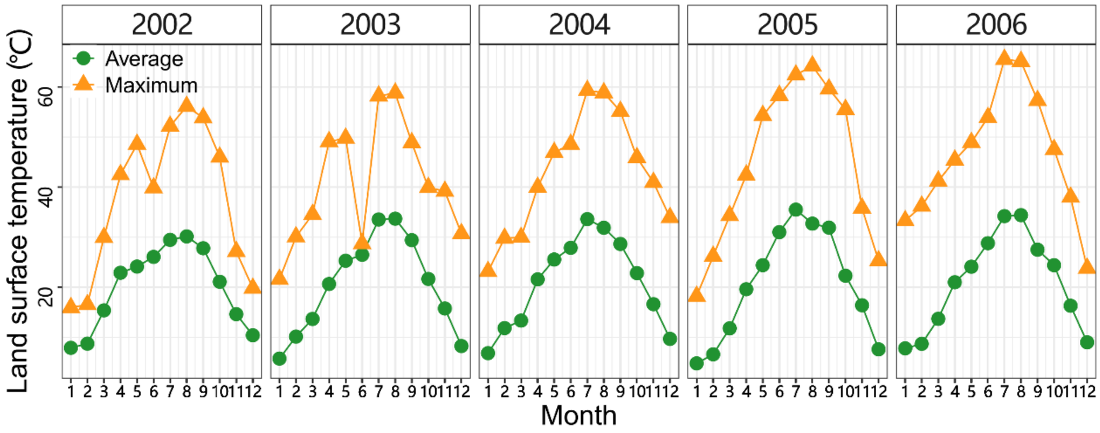

Figure S1. Average and maximum land surface temperature of the sampling site spanning the period from 2002 to 2006. The data were provided by the National Ecosystem Science Data Center, National Science & Technology Infrastructure of China (http://www.nesdc.org.cn).

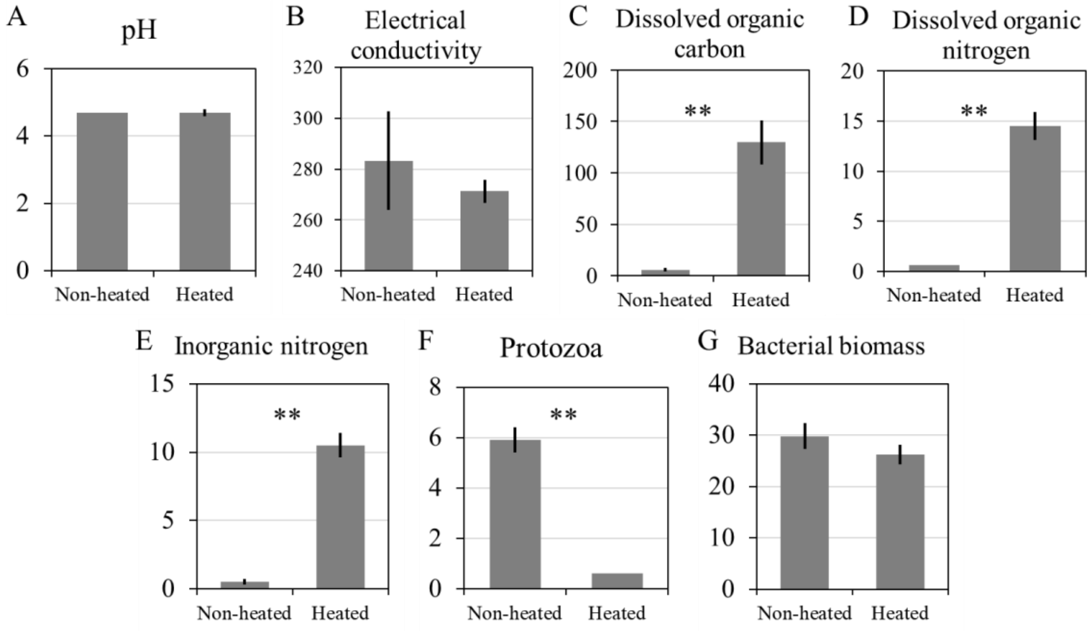

Figure S2. Soil physicochemical and bacterial properties, including pH, electrical conductivity (us/cm), dissolved organic carbon (mg kg^{-1}), dissolved organic nitrogen (mg kg^{-1}), inorganic nitrogen (mg kg^{-1}), protozoa (log cells g^{-1}), and bacterial biomass (nmol g^{-1}), in non-heated and heated soils before inoculating the non-indigenous bacteria (mean ± SD). The pair-wise two-sample *t*-test was employed between disturbed and non-disturbed soils. *$P < 0.05$ and ** $P < 0.01$.
